# Solvent-Free Method of Polyacrylonitrile-Coated LLZTO Solid-State Electrolytes for Lithium Batteries

**DOI:** 10.3390/molecules29184452

**Published:** 2024-09-19

**Authors:** Xuehan Wang, Kaiqi Zhang, Huilin Shen, Hao Zhang, Hongyan Yao, Zheng Chen, Zhenhua Jiang

**Affiliations:** Engineering Research Center of Special Engineering Plastics, Ministry of Education, National and Local Joint Engineering Laboratory for Synthetic Technology of High Performance Polymer, College of Chemistry, Jilin University, Qianjin Street 2699, Changchun 130012, China; xuehan23@mails.jlu.edu.cn (X.W.);

**Keywords:** composite solid-state electrolytes, solvent-free method, high ionic conductivity

## Abstract

Solid-state electrolytes (SSEs), particularly garnet-type Li_6.4_La_3_Zr_1.4_Ta_0.6_O_12_ (LLZTO), offer high stability and a wide electrochemical window. However, their grain boundaries limit ionic conductivity, necessitating high-temperature sintering for improved performance. Yet, this process results in brittle electrolytes prone to fracture during manufacturing. To address these difficulties, solvent-free solid-state electrolytes with a polyacrylonitrile (PAN) coating on LLZTO particles are reported in this work. Most notably, the PAN-coated LLZTO (PAN@LLZTO) electrolyte demonstrates self-supporting characteristics, eliminating the need for high-temperature sintering. Importantly, the homogeneous polymeric PAN coating, synthesized via the described method, facilitates efficient Li^+^ transport between LLZTO particles. This electrolyte not only achieves an ionic conductivity of up to 2.11 × 10^−3^ S cm^−1^ but also exhibits excellent interfacial compatibility with lithium. Furthermore, a lithium metal battery incorporating 3% PAN@LLZTO-3%PTFE as the solid-state electrolyte and LiFePO_4_ as the cathode demonstrates a remarkable specific discharge capacity of 169 mAh g^−1^ at 0.1 °C. The strategy of organic polymer-coated LLZTO provides the possibility of a green manufacturing process for preparing room-temperature sinter-free solid-state electrolytes, which shows significant cost-effectiveness.

## 1. Introduction

In recent years, as a representative of the energy industry, the scale of lithium batteries [1,2] is constantly expanding. At present, the cathodes of automobile batteries, both at domestic and international levels, are mainly LiFePO_4_ [3] and LiCoO_2_ [4], etc. Nevertheless, most of these batteries have reached their highest theoretical energy density and suffer from safety risks. Solid-state batteries (SSBs) [5,6] are considered to be one of the most promising categories, replacing the organic electrolyte and diaphragm in existing lithium batteries with solid-state electrolytes (SSEs) [7,8]. These batteries are noted for their enhanced cycle life, compact form factor, scalability, adaptability of design, and straightforward packaging, which are attributes that align with the progressive evolution of energy storage solutions. 

SSBs are differentiated based on their electrolyte composition into two primary categories: organic and inorganic. Organic solid-state electrolytes are typified by polyethylene oxide (PEO) [9,10], polyacrylonitrile (PAN) [11,12,13,14], etc. Inorganic solid electrolytes are mainly characterized by oxides and sulfides, etc. The prevalent electrolytes in SSBs are oxides, sulfides, and polymers, with oxide electrolytes exhibiting a better comprehensive performance and substantial developmental prospects. Oxide solid electrolytes can be classified into five principal types: (1) garnet (e.g., Li_6.4_La_3_Zr_1.4_Ta_0.6_O_12_, LLZTO) [15,16,17,18], (2) Lithium-ion Conductor (LISICON, e.g., Li_14_Zn (GeO_4_)_4_) [19], (3) Lithium ion-containing Na^+^ Super Ionic Conductor (NASICON, e.g., Li_2_Ti_2_(PO_4_)_3_) [20], (4) perovskite (e.g., Li_0.33_La_0.55_TiO_3_), and anti-perovskite (e.g., Li_2_OHCl). Among these, the garnet type stands out for its higher ionic conductivity, robust lithium metal stability, and significant long-term developmental potential [21,22]. In particular, LLZTO has the advantages of Li friendliness, high electrochemical stability, and a wide electrochemical window (0–6 V vs. Li^+^/Li) [23].

Current research underscores significant advancements in the mass production and market adoption of garnet electrolytes. Notably, Wachsman’s group [24] focused on industrial-scale applications, endeavors to realize high-capacity, high-energy-density solid-state lithium–sulfur batteries via a cost-effective strategy. By eliminating the impurity phase from the Ta-LLZTO surface, they have created batteries with a runtime exceeding 300 days and a capacity retention rate of 80%. As another example, Guo’s research group [25] has introduced LLZTO with ceramic particles coated by a polyacrylonitrile (PAN) layer, synthesized via a flow-assisted method. This innovation has resulted in the diminished interfacial impedance between garnet ceramics and lithium metal, concurrently enhancing the electrochemical stability of the developed solid-state batteries. However, LLZTO has several critical limitations in research and practical applications. (1) Environmental sensitivity: The surface of LLZTO can easily generate lithium-hating impurities, e.g., LiOH and Li_2_CO_3_ [26], which are harmful to the stable cycling of batteries. (2) Insufficient softness: LLZTO solid-state electrolytes do not have enough “softness” to adapt to the deformation strain of the electrode material, although the isotropic shear modulus (60 GPa) [27] is higher than the critical value required to suppress lithium dendrites (8.5 GPa) [28]. (3) High application requirements: Unlike sulfide-based solid electrolytes, LLZTO particles cannot support fast inter-particle Li^+^ conduction [29] at loosely stacked grain/particle boundaries and generally need the additional work of sintering the particles or applying external pressure. (4) Difficulty in production: Due to the high-hardness behavior of LLZTO SSEs (Young’s modulus ≈ 150 GPa, Bulk modulus ≈ 100 GPa), electrolyte membranes with a thickness of less than 200 μm [30] are tough to produce. The above series of problems seriously prevent the usage of LLZTO SSEs. Therefore, it is necessary to overcome the obstacle of the heterogeneous phase on the SSEs’ surface, high hardness, harsh preparation conditions, and processing. Currently, novel LLZTO SSEs materials with both excellent Li^+^ conductivity and simple fabrication and application urgently need to be developed.

In this study, we coated 3% homogeneous PAN on the surface of LLZTO particles. The close chemical interactions at the PAN/LLZTO interface contribute to inducing partial de-hydrocyanation of PAN and result in a localized conjugated structure, which can benefit the transport of Li^+^ among 3% PAN@LLZTO particles. Nevertheless, the enhancement of high safety and energy density (>500 wh kg^−1^) batteries [31] is difficult to achieve with only a single material. We discovered that the solvent-free method [32,33,34,35] used to prepare electrode films is also suitable for the preparation of LLZTO SSEs membranes. This simple and energy-efficient preparation method requires only a small percentage of polytetrafluoroethylene binder without a solvent. Controlled mixing, microfibrillation, and fibrillation steps could be employed to repeatedly calendar 3% PAN@LLZTO particles to obtain thin electrolyte membranes. Finally, we observed that electrolyte membranes (≈50 μm) prepared by biaxial calendaring at 80 °C using 3%PTFE can achieve an ionic conductivity of >10^−3^ S cm^−1^ without sintering or applying external pressure. Notably, we used a lithium metal battery with 3% PAN@LLZTO-3%PTFE as the SSEs and LiFePO_4_ as the cathode exhibits an excellent discharge-specific capacity (169 mAh g^−1^) at 0.1 °C. The investigation into solvent-free composite solid-state electrolytes (SSEs) represents a seminal advancement in the field, enabling the feasibility of sinter-free SSEs at room temperature. This sophisticated methodology is poised to redefine the frontiers of cost-effectiveness within the domain of energy storage technologies.

## 2. Results and Discussion

**Figure 1:**
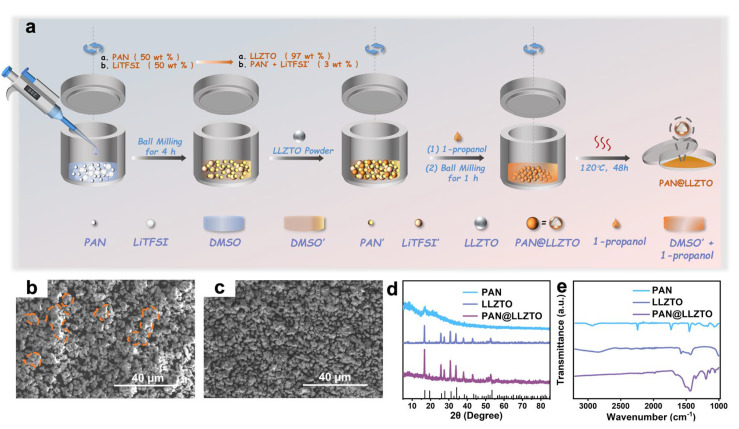
(**a**) Synthesis of 3% PAN@LLZTO composite electrolyte powder; SEM images: (**b**) LLZTO powder and (**c**) 3% PAN@LLZTO powder; (**d**) XRD image and (**e**) FT-IR spectra of PAN, LLZTO, and 3% PAN@LLZTO.

The synthesis of 3% PAN@LLZTO composite electrolyte powder was carried out by the solution precipitation method, and the specific process is shown in Figure 1a (refer to the Experimental Section). Keeping a uniform distribution of LLZTO particles in the polymer network is crucial. Scanning electron microscope (SEM) images of LLZTO and 3% PAN@LLZTO powders are shown in Figure 1b,c. The electrostatic interaction between PAN, LLZTO, and DMSO occurs during the synthesis process, which can promote the even distribution of the polymer coating on the surface of the particles and can prevent the aggregation of LLZTO particles [25]. Therefore, PAN has significant advantages as a coating material for LLZTO. As shown in the XRD image (Figure 1d), the 3% PAN@LLZTO powder did not undergo a chemical change compared to the LLZTO. This result illustrates that the PAN surface coating cannot change the crystal structure of LLZTO.

In the FT-IR spectra (Figure 1e), the peak intensities of 3% PAN@LLZTO at 2936 cm^−1^ (stretching vibrational mode of the C-H bond) and 2242 cm^−1^ (stretching vibrational mode of the -CN bond) are obviously lower than PAN. The observation of a peak at 2212 cm^−1^, which we hypothesize is indicative of the formation of a novel compound LiCN, suggests that the polyacrylonitrile (PAN) may have experienced a dehydrogenation reaction. Notably, pure PAN is characterized by a white color, in contrast to the brownish-yellow appearance of PAN-coated LLZTO, as depicted in Figure S1a,b. The interaction between DMSO and LLZTO is believed to induce a redistribution of electron density within the DMSO molecule, thereby promoting the dehydrogenation of PAN on the LLZTO surface and leading to the emergence of a conjugated structure [25]. Additionally, the introduction of pure DMSO to PAN and LiTFSI results in a light-yellow coloration, as illustrated in Figure S2a,b, with the hue becoming more pronounced in the presence of both DMSO and LLZTO. It should be noted that the addition of LiTFSI is solely intended to enhance the ionic conductivity of the solid-state electrolyte, and it does not react chemically. These color changes, alongside the results from the FT-IR spectroscopy, collectively support the inference of a dehydrogenation reaction taking place. Furthermore, when exposed to air, LLZTO is prone to the formation of alkaline surface impurities, including LiOH and Li_2_CO_3_. Interestingly, LiOH has been identified as a catalyst for the de-hydrocyanation of PAN [36]. As a result, the modification of LLZTO with the ionic conductor PAN is posited as a multifaceted strategy. This approach not only enhances the conductivity of Li^+^ at the ceramic/polymer interface but also contributes to the mitigation of surface contamination on LLZTO.

**Figure 2:**
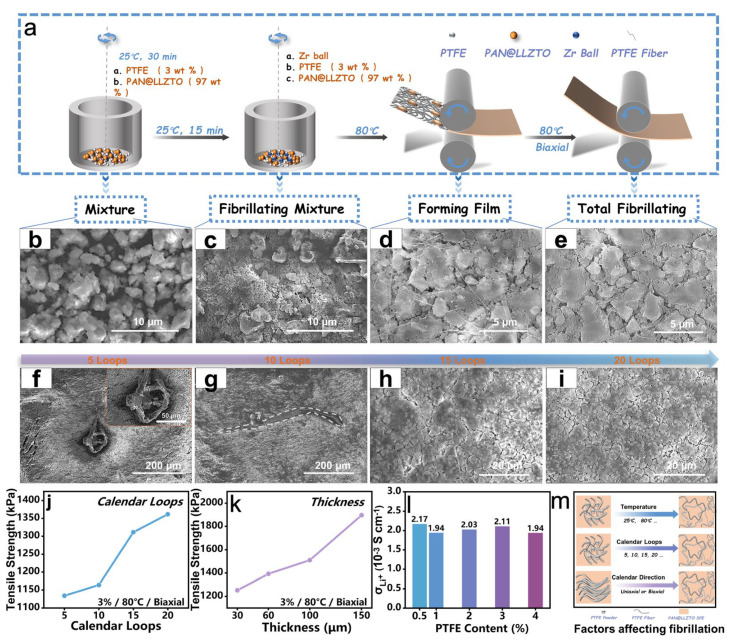
(**a**) Synthesis of 3% PAN@LLZTO-PTFE membranes; SEM of: (**b**) the mixture of 3% PAN@LLZTO and PTFE, (**c**) 3% PAN@LLZTO powder and micro-fibrillated PTFE flocculent, (**d**) enhanced fibrillation of the flocculent structure, (**e**) total fibrillating of the 3% PAN@LLZTO-PTFE; SEM of 3% PAN@LLZTO-PTFE calendaring after: (**f**) 5 times; (**g**) 10 times; (**h**) 15 times; (**i**) 20 times; Variation of (**j**) tensile strength with the number of calendaring cycles, (**k**) tensile strength with electrolyte film thickness, and (**l**) ionic conductivity with PTFE content; (**m**) factors influencing the fibrillation degree of solvent-free SSE films.

The preparation of the SSEs membrane by the 3% PAN@LLZTO powder is shown in the preparation process in Figure 2a. Pre-mixing, microfibrillating, and fibrillating LLZTO with commercial PTFE followed by calendaring can yield 3% PAN@LLZTO-PTFE membranes. The specific procedure was as follows: Firstly, the 3% PAN@LLZTO electrolyte and PTFE particles were uniformly mixed using a ball mill without zirconium balls for 15 min in Figure 2b. Subsequently, the mixed powder described above was microfibrillated in a ball mill with zirconium balls added for 5 min to obtain a flocculated PAN@LLZTO-PTFE matrix in Figure 2c. Finally, the above-obtained flocculent was calendared with a roller press to a defined thickness, which provided an enhancement of the fibrillation in Figure 2d and allowed the fibers to be clearly seen. After that, all the LLZTO particles were distributed in the tightly connected PTFE fibers to obtain a solvent-free solid electrolyte membrane of 3% PAN@LLZTO-PTFE. The top view of the PAN@LLZTO-PTFE membrane shows complete fibrosis in Figure 2e. The FT-IR spectra (Figure S3) of the PAN@LLZTO powder and PAN@LLZTO-PTFE membrane show a significant shift compared to the PTFE in the bands at 1207 and 1151 cm^−1^. It proves that PTFE and LLZTO form interactions during the preparation process.

In this research, we have conducted an in-depth investigation into the factors influencing the fabrication of PTFE-LLZTO electrolyte films. Scanning electron microscopy (SEM) was employed to analyze the microstructural alterations of the materials, as depicted in Figure 2f–i. SSEs membranes were fabricated using varying calendaring times (5, 10, 15, and 20), while maintaining consistent PTFE content. The PAN@LLZTO-PTFE samples subjected to 5 and 10 cycles of calendaring exhibited a fibrillated structure (Figure 2f,g), with distinct PAN@LLZTO and microfibrillated PTFE phases being discernible. SEM images (Figure 2h,i) reveal that the tensile strength of the PAN@LLZTO-PTFE composites increase with the number of calendaring cycles as the surface becomes more compact. In Figure 2j, there is a significant enhancement in the tensile strength of the SSEs film when the calendaring cycles reach 15 times (288 kPa) or more. This finding suggests a correlation between the number of calendaring cycles and the extent of fibrillation. However, the degree of fibrillation is not solely dependent on the number of calendaring cycles; it was also observed that the tensile strength of the SSEs membranes increases with thickness when thickness is the sole variable (Figure 2k). It is important to note that variations in PTFE content can fundamentally affect the degree of fibrillation, which subsequently impacts the intimate contact between the PAN@LLZTO particles, as reflected in the differences in Li^+^ conductivity. Although the PTFE binder is capable of binding LLZTO particles to form membranes, the incorporation of excessive insulating PTFE may diminish the Li^+^ conductivity of the resulting membranes. This underscores the delicate balance required in the formulation of PTFE-LLZTO electrolyte films to optimize both mechanical strength and ionic conductivity.

To investigate the influence of PTFE content on the electrochemical behavior of SSE membranes, SS|PAN@LLZTO-PTFE|SS were assembled with PTFE membranes of uniform thickness (60 ± 2 μm). The Li^+^ conductivity of the SSE membranes was determined from the electrochemical impedance spectra (EIS). In Figure 2l, although the highest ionic conductivity (2.17 × 10^−3^ S cm^−1^) was observed at a PTFE ratio of 0.5%, the resulting SSE membrane structure was too loose for practical application. Consequently, the SSE membrane with the next highest ionic conductivity was selected, which, at a PTFE content of 3%, exhibited an ionic conductivity of 2.11 × 10^−3^ S cm^−1^. In contrast, an excessive PTFE content, such as 4%, resulted in a decrease in ionic conductivity. The optimal PTFE content facilitates close contact between LLZTO particles, thereby maximizing Li^+^ conductivity. For all electrochemical studies in this work, PAN@LLZTO membranes prepared with 3% PTFE (3% PAN@LLZTO-3%PTFE) were utilized. Furthermore, the fibrillation of SSE membranes must account for the effects of temperature and calendaring direction. Given that the glass transition temperature (Tg) of PTFE exceeds 80 °C [37], all SSE membranes were calendared at this temperature in the current study. Unidirectional calendaring tends to create aggregated one-dimensional (X direction or Y direction) fibers, whereas biaxial-directional (X direction and Y direction) calendaring creates randomly dispersed fibers. Hence, biaxial-directional calendaring was employed throughout this work. In conclusion, various conditions, including PTFE content, calendaring frequency, film thickness, temperature, and calendaring direction, affect the fibrillation degree of solvent-free SSE membranes, as illustrated in Figure 2m. Therefore, to enhance the physical and electrochemical properties of SSE membranes, a multifaceted consideration [38] of these conditions is necessary, rather than focusing on a single factor.

**Figure 3:**
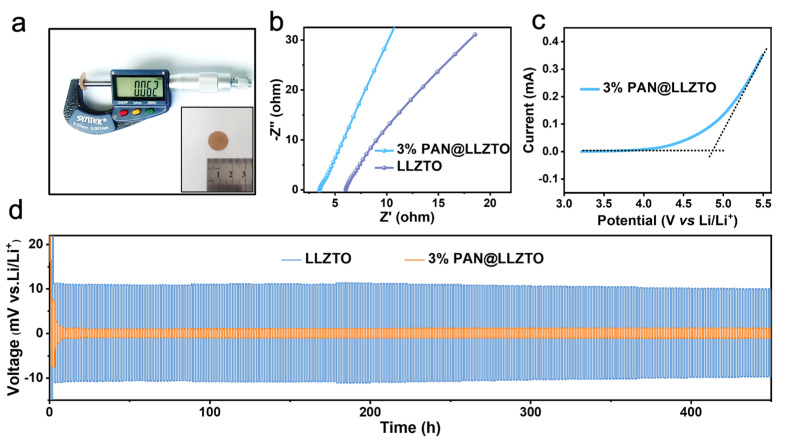
(**a**) Thickness measurement diagram and schematic diagram for cutting 16mm diameter of 3% PAN@LLZTO-3%PTFE; (**b**) impedance of LLZTO-3%PTFE and 3% PAN@LLZTO-3%PTFE; (**c**) linear scanning voltammetry of 3% PAN@LLZTO-3%PTFE; (**d**) Li plating/stripping curves of LLZTO-3%PTFE and 3% PAN@LLZTO-3%PTFE.

The 60 ± 2 μm membranes (Figure 3a) used in the EIS tests, (Figure 3b) as well as the SS|LLZTO-3%PTFE|SS and SS|3% PAN@LLZTO-3%PTFE|SS, were of uniform thickness. The impedance of the 3% PAN@LLZTO-3%PTFE membrane was found to be significantly lower (3.48 Ω vs. 6.04 Ω), signifying its superior Li^+^ conductivity. This enhanced ionic conductivity is beneficial in mitigating concentration polarization during the battery’s charge and discharge cycles. The operational voltage of a battery is a critical parameter that affects its performance. To assess the electrochemical stability of the electrolyte, linear scanning voltammetry (LSV) was employed on the cell SS|3% PAN@LLZTO-3%PTFE|Li. The LSV curve shows a horizontal shape followed by a rising curve. The finding in Figure 3c indicates that this electrolyte can support normal battery operation up to a maximum voltage of approximately 4.9 V versus Li/Li^+^. The electrolyte membrane fabricated in this study, 3% PAN@LLZTO-3%PTFE, demonstrated excellent interfacial compatibility with lithium metal.

To further evaluate the dynamic stability of lithium ions at the interface between 3% PAN@LLZTO-3%PTFE and lithium metal, a Li|3% PAN@LLZTO-3%PTFE|Li battery was subjected to the plating and stripping process at a current density of 0.1 mA cm^−2^. The experimental outcomes, as shown in Figure 3d, reveal that these lithium cells were capable of cycling stably for over 450 h with a maintained overpotential. Additionally, the overpotential of the Li|3% PAN@LLZTO-3%PTFE|Li was notably lower (±1.3 mV) than that of the Li|LLZTO-3%PTFE|Li (±11 mV). In addition, we summarize recent representative work on solvent-free inorganic solid-state electrolytes in Table 1. Comparisons were made in terms of thickness, PTFE content, and ionic conductivity, respectively. The comparison reveals that the electrolyte films prepared in this work have the highest conductivity and have potential for development.

**Figure 4:**
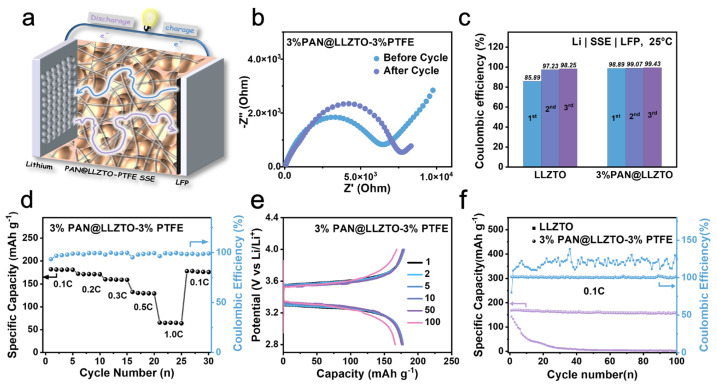
Electrochemical properties of LFP|3% PAN@LLZTO-3%PTFE|Li batteries. (**a**) Battery’s structure diagram; (**b**) impedance before and after cycle; (**c**) Coulombic efficiency during the three cycles; (**d**) discharge rate curves at 0.1 °C, 0.2 °C, 0.3 °C, 0.5 °C, 1.0 °C; (**e**) charge and discharge voltage profiles obtained at different cycles; (**f**) long-term cycling performance at 0.1 °C compared with LFP|LLZTO-3%PTFE|Li.

Since 3% PAN@LLZTO has excellent Li^+^ conductivity and lithium metal compatibility, it can be prepared as a solvent-free SSE (3% PAN@LLZTO-3%PTFE) and then assembled with a Li anode and LiFePO_4_ (LFP) cathode (Figure 4a). The EIS test was performed at 25 °C for the battery before cycling and after 100 cycles. Nyquist plots captured from 1 Hz to 1 MHz at open circuit potential revealed a high-frequency semicircle, correlating to charge-transfer resistance (R_ct_). [40,41] As depicted in Figure 4b, R_ct_ increased from 6.25 × 10^3^ to 7.7 × 10^3^ Ω post charge–discharge cycle. Figure 4c shows the Coulombic efficiency of the LFP|3% PAN@LLZTO-3%PTFE|Li during the three cycles, and the values are all better than those of the LFP|LLZTO-3%PTFE|Li. In particular, the Coulombic efficiency of the 3% PAN@LLZTO-3%PTFE half-cell for the first cycle is close to 99%, indicating that its cycling reversibility is excellent. In addition, in the multiplicity test curve (Figure 4d), the discharge capacity of this cell was as high as 169 mAh g^−1^ at 0.1 °C, nearly close to its theoretical capacity of 170 mAh g^−1^. As the current was increased from 0.1 °C to 1.0 °C and then restored to 0.1 °C, the reversible capacities of the electrodes were 70 and 159 mAh g ^−1^, respectively. Furthermore, the cyclic reversibility of the LFP|3% PAN@LLZTO-3%PTFE |Li charge–discharge performance at 1, 2, 5, 10, 50, and 100 cycles can be seen in Figure 4e. The charge–discharge curves overlap very well, indicating the excellent reversibility, as well as the stable performance of the batteries. The capacity retention of the 3% PAN@LLZTO-3%PTFE batteries (92.8%), was significantly higher than that of the LLZTO-3%PTFE half-batteries (3.4%) for 100 cycles at 0.1 °C (Figure 4f), and this is probably attributed to the excellent Li^+^ conductivity of the electrolyte. Based on the above electrochemistry of the batteries, the 3% PAN@LLZTO-3%PTFE synthesized using the solvent-free production method proposed herein holds great promise for improving the performance of lithium metal batteries.

## 3. Materials and Methods

### 3.1. Materials

Polyacrylonitrile (PAN) was purchased from Shanghai Aladdin Biochemical Technology Co., Ltd. Li_6.4_La_3_Zr_1.4_Ta_0.6_O_12_ (LLZTO) was purchased from Shenzhen Neware Co., Ltd. 1-methyl-2-pyrrolidinone (NMP) (99.7%), methyl sulfoxide (DMSO), and 1-propanol were purchased from Sigma-Aldrich (Shanghai) Trading Co., Ltd. LiFePO_4_ (LFP), Super P, Li foil (∅ 15.6 mm, thickness: 0.45 mm), and electrolyte (1 M LiPF_6_ in EC:DEC = 1:1 vol% with 5 vol% FEC) were purchased from Dongguan Canrud Innovative Technology Co., Ltd. PTFE binding was purchased from Daikin Fluorochemical (China) Co., Ltd.

### 3.2. Preparation of 3% PAN@LLZTO Powder

Initially, PAN and lithium bis(trifluoroethane)sulfonamide salt (LiTFSI) was dissolved in methyl sulfoxide (DMSO) in a mass ratio of 1:1 and ball-milled for 4 h. Subsequently, under an argon atmosphere, LLZTO was added in the mass ratio of LLZTO: PAN = 100:3 and then continued to be ball-milled for 12 h. Finally, 1-propanol was added to the above mixture and ball-milled for 1 h. The mixture was dried under a vacuum at 80 °C for 48 h to remove the solvent. The obtained 3% PAN@LLZTO powder was stored in an argon-filled glove box.

### 3.3. Preparation of PTFE-LLZTO Electrolyte Membrane

First, the 3% PAN@LLZTO electrolyte and PTFE particles were homogeneously mixed using a ball mill without zirconium balls for 15 min at room temperature. Subsequently, the above-mixed powder obtained was microfibrillated in a ball mill grinder with the addition of zirconium balls for 5 min to obtain a flocculent 3% PAN@LLZTO-PTFE matrix. Finally, the above matrix obtained was calendared to a specified thickness by a roller press to obtain a solvent-free solid electrolyte membrane of 3% PAN@LLZTO-PTFE.

### 3.4. Preparation of LFP Electrodes and Cell Assembling

The LFP cathode was prepared by mixing LFP, Super P, PVDF, LiTFSI, and PAN with a mass ratio of 70:10:10:5:5. The slurry was coated on aluminum foil and dried at 80 °C for 10 h. The electrolyte was controlled at 15 μL. The electrolyte tends to minimize the poor contact at the electrolyte/electrode interface and to avoid direct failure of the battery to operate. The mass loading of LFP was controlled within 1–1.5 mg cm^−2^. All coin cells were assembled with designed electrodes and electrolytes in a glove box (H_2_O and O_2_ ≤ 0.01 ppm).

### 3.5. Characterizations and Electrochemical Measurements

A Fourier transform infrared (FT-IR) spectrometer (Bruker Tensor-27, Saarbrucken, Germany) was used to examine the chemical structures. X-ray diffraction (XRD) patterns were collected on a Rigaku X-ray diffractometer (D/max 2500, Rigaku, The Woodlands, TX, USA) to identify the crystal structure. A scanning electron microscope (SEM) was used to visualize the morphology and elemental distribution (JEOL JSM-6701F, JEOL Ltd., Tokyo, Japan). Electrochemical measurement (electrochemical workstation of CHI-660E, Neware battery test system, CT-4008T-5V10mA-164, Shenzhen, China).

## 4. Conclusions

In summary, we report a solvent-free solid electrolyte membrane prepared from a polymer PAN-coated oxide electrolyte. The modification of LLZTO with an ionic conductor PAN not only contributes to the enhancement of Li^+^ conductivity at the ceramic/polymer interface but also supports the removal of contaminants from the LLZTO surface. In addition, we tested the tensile strength of SSEs prepared with different process parameters (content of PTFE, number of calendaring, film thickness, temperature, and calendaring direction). The 3% PAN@LLZTO-3%PTFE prepared using this strategy has good compatibility with Li metal and excellent Li^+^ conductivity. The electrochemical performance of LFP|Li half-cells assembled with 3% PAN@LLZTO-3%PTFE was much higher than that of LLZTO-3%PTFE without PAN modification. Therefore, the present work is feasible and tremendously cost-effective for facilitating the transition of SSB technology from the laboratory to the factory.

## Figures and Tables

**Table 1:** Electrochemical properties of lithium-based batteries using solvent-free electrolytes membrane.

Solvent-Free Membrane	Thickness (µm)	PTFE (wt%)	Ionic Conductivity (10^−3^ S cm^−1^) 25 °C	Batteries	References
Li_3_InCl_6_/PTFE	20	0.5	1.00	Li_3_InCl_6_@LiCoO_2_|Li_3_InCl_6_/LPSC|Graphite/LPSC	[39]
LPSC/S/C/PTFE	93	1.0	0.63	S/C/LPSC|LPSC|Li	[32]
LPSC/PTFE	300	2.0	1.28	NCM811/LPSC|LPSC|Si	[37]
LLZTO/PTFE	20	0.5	0.52	NCM811|LLZTO/LiTFSI in DMSO with 10% FEC|Si-C-450	[39]
LLZTO/PTFE	20	3.0	0.66	LFP|LLZTO/LiPF_6_ in EC:PC = 1:1 vol% with 5% FEC|Li	[38]
PAN@LLZTO/PTFE	62	3.0	2.11	LFP|PAN@ LLZTO/LiPF_6_ in EC:DEC = 1:1 vol% with 5% FEC|Li	This work

LPSC (Li_6_PS_5_Cl), NCM811 (LiNi_0.8_Mn_0.1_Co_0.1_O_2_), LLZTO (Li_6.4_La_3_Zr_1.4_Ta_0.6_O_12_), LFP(LiFePO_4_).

## Data Availability

Data are available in the source publications listed in the bibliography.

## Supplementary Materials

The following supporting information can be downloaded at https://www.mdpi.com/article/10.3390/molecules29184452/s1. Figure S1: PAN powder and 3% PAN@LLZTO powder; Figure S2: color of DMSO/PAN and DMSO/PAN/LLZTO; Figure S3: FT-IR of PAN, PAN@LLZTO, and PAN@ LLZTO-PTFE membrane.
