# Supplementary material for: Solvent-Free Method of Polyacrylonitrile-Coated LLZTO Solid-State Electrolytes for Lithium Batteries
Source: Molecules. 2024 Sep 19;29(18):4452. doi: 10.3390/molecules29184452 (PMC11434599; doi:10.3390/molecules29184452)
Supplement: Supplementary file 1 [file molecules-29-04452-s001.zip › molecules-3179078-supplementary.pdf]

Supporting Information

Solvent-Free Method of Polyacrylonitrile-Coated LLZTO Solid-State Electrolytes for Lithium Batteries

Xuehan Wang [1], Kaiqi Zhang [1], Huilin Shen [1], Hao Zhang [1], Zheng Chen [1]*, and Zhenhua Jiang [1]

[1]Key Laboratory of High-Performance Plastics, Ministry of Education, National & Local Joint Engineering Laboratory for Synthesis Technology of High-Performance Polymers, College of Chemistry, Jilin University, Xiuzheng Road 1788, Changchun 130012, China

*Corresponding authors.

Email address: chenzheng2013@jlu.edu.cn (Z.C.)

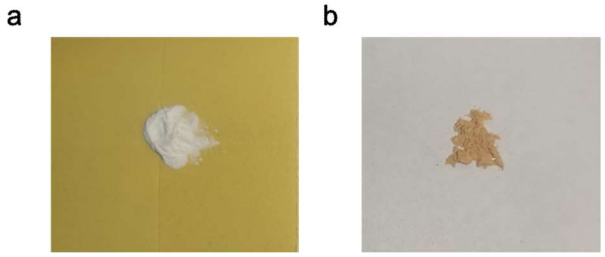

Figure S1. (a) PAN powder and, (b) 3%PAN@LLZTO powder.

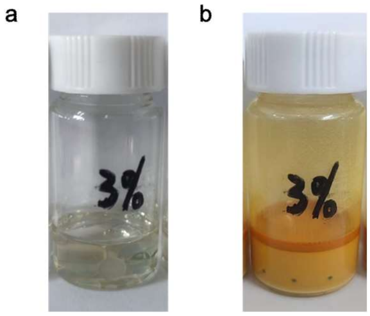

Figure S2: (a) DMSO/PAN, (b)DMSO/PAN/LLZTO' color.

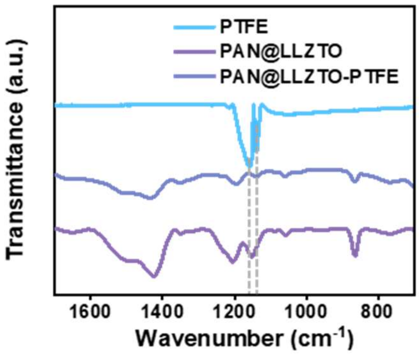

Figure S3: FT-IR of PAN, PAN@LLZTO, and PAN@ LLZTO-PTFE membrane.
